# Antimicrobial properties of CuO nanorods and multi-armed nanoparticles against *B. anthracis* vegetative cells and endospores

**DOI:** 10.3762/bjnano.5.91

**Published:** 2014-06-05

**Authors:** Pratibha Pandey, Merwyn S Packiyaraj, Himangini Nigam, Gauri S Agarwal, Beer Singh, Manoj K Patra

**Affiliations:** 1 Electron Microscopy Division Defence R&D Establishment, New Campus, Jhansi Road, Gwalior, India; 2 Microbiology Division Defence R&D Establishment, New Campus, Jhansi Road, Gwalior, India; 3 Protective Devices Division Defence R&D Establishment, New Campus, Jhansi Road, Gwalior, India; 4 Defence Laboratory, Jodhpur, India

**Keywords:** *Bacillus anthracis*, bactericidal nanoparticles, copper oxide, nanoparticles, spore inactivation

## Abstract

Two different kinds of CuO nanoparticles (NPs) namely CuO nanorods (PS2) and multi-armed nanoparticles (P5) were synthesized by wet and electrochemical routes, respectively. Their structure, morphology, size and compositions were characterized by SEM, EDX and XRD. The NPs demonstrated strong bactericidal potential against *Bacillus anthracis* cells and endospores. PS2 killed 92.17% of 4.5 × 10^4^ CFU/mL *B. anthracis* cells within 1 h at a dose of 1 mg/mL. Whereas P5 showed a higher efficacy by killing 99.92% of 7 × 10^5^ CFU/mL *B. anthracis* cells within 30 min at a dose of 0.5 mg/mL and 99.6% of 1.25 × 10^4^ CFU/mL *B. anthracis* cells within 5 min at a dose of 2 mg/mL. More than 99% of spores were killed within 8 h with 2 mg/mL PS2 in LB media.

## Introduction

*B. anthracis,* the etiological agent of anthrax is a gram-positive, rod shaped, spore forming bacterium with 1 to 8 µm length and 1 to 1.5 µm width [1]. Its very high lethality (LD_50_ is 2,500 to 55,000 spores from inhalation route) [1], easy production, environmental stability of spores and their easy dissemination through aerosolization makes it one of the most important biological warfare agent and bioterrorism threats. Dormant *B. anthracis* spores are highly resistant to adverse environmental conditions. The spores can survive for prolonged periods in soil despite extremes of temperature, desiccation, chemical treatment and UV exposure [2–6]. The possible development and release of genetically engineered strains that are resistant against antibiotics and vaccines, similar to those developed by the former Soviet Union, is another challenge [6].

Disinfectants like formaldehyde, glutaraldehyde, phenols, ethylene oxide, chlorine dioxide, peracetic acid, sodium hypochlorite etc. show high inactivation effect against *B. anthracis* spores. However, the generation of toxic fumes, the carcinogenicity and the corrosive nature limits their use for personal decontamination and decontamination of sensitive equipment [7]. This scenario makes it imperative to evaluate antimicrobial potential of the robust nanoparticles. Active noncorrosive nanoparticles can be used for disinfection of equipment and surfaces for example, in air filters and respirators and also, in paints and coatings for hospitals and strategic buildings.

Antimicrobial formulations comprising inorganic nanoparticles could be effective bactericidal materials. They have the additional advantage of improved safety and stability of inorganic agents compared to organic antimicrobial agents [8]. The safe and efficient decontamination of civilian water resources and facilities after an attack with *B. anthracis* is a potential application for nanomaterials. A considerable amount of research was published recently in the field of nanoparticle based deactivation of microbes for medical and healthcare applications with nano-silver taking lead in academic research as well as in commercialization [9–12]. Comparatively few reports are available on the evaluation of nanoparticles against biological warfare (BW) agents including deadly *B. anthracis* spores and vegetative cells. However the limited work still underscores the potential of a nanomaterial-mediated decontamination of BW agents. Prasad et al. [13] have reported some degree of deactivation of *B. anthracis* cells by UV light assisted TiO_2_ nanoparticles at a dosage ranging from 10 to 100 mg per 2000 cells. Haggstrom et al. [14] have reported sporicidal activity of corrosive halogen adducts of nanometer-scaled active metal oxides Al_2_O_3_, TiO_2_ and CeO_2_ in a solid based interaction on microfiltration membranes. The ratio of bacterial agents to metal oxide is not clear from this particular reference, but in an earlier publication the group reported the use of 250 mg APMgO nanoparticles against 10^6^ cells in a similar study [15]. As the method described by Haggstrom et al. is identical with the previous work [15], we expect similar amounts (about 250 mg) of nanometer-scaled oxides have been used in the later work as well. However, the application of corrosive chemicals along with the nanoparticles indicates their limited utility for decontamination of indoors, sensitive equipment and surfaces.

Recently we reported strong bactericidal activity of nanometer-scaled CuO against a large number of gram-positive and gram-negative bacteria [16]. CuO nanostructures were reported as potential antibacterial agents by other groups as well [17–20]. Trapalis et al. [17] and Akhavan et al. [18] reported CuO–SiO_2_ composite thin film and CuO/Cu(OH)_2_ nanostructure, respectively, generated on copper foil as effective antibacterial against *E. coli* bacteria when the bacterial suspension drop was tested on these surfaces. Perelshtein et al. [19] have reported antibacterial CuO-cotton textile against *E. coli* and *S. aureus*. Gao et al. [20] reported strong antibacterial activity of CuO nanostructures comparable to established antibiotics as well as their photocatalytic potential. However, we have not come across any report on bactericidal potential of CuO nanoparticles against *B. anthracis* cells and spores. The earlier findings inspired us to evaluate antibacterial activity of noncorrosive CuO nanoparticle against *B. anthracis*. As *B. anthracis* bacteria can exist in vegetative cell state or in dormant spore state, killing both forms is important for an effective decontamination. In this study we have synthesized nanometer-scaled CuO in two different morphologies, one rod shaped and the other multi-armed nanoparticles. Both were evaluated for bactericidal and sporicidal activity against *B. anthracis* Sterne vegetative cells and spores.

CuO nanorods (PS2) were synthesized by a novel wet-chemical route at room temperature in aqueous solution. CuO multi-armed nanoparticles (P5) were synthesized by an electrochemical route [16]. The nanoparticles were characterized by SEM/EDX and XRD for formation, structure, morphology, composition and phase determination as well as for antibacterial efficacy. CuO nanorods (PS2) and CuO multi-armed nanoparticles (P5) both have demonstrated excellent bactericidal efficacy against gram-positive *B. anthracis* vegetative cells almost comparable to that against nonsporigenic gram-negative *E. coli* bacteria. The CuO nanoparticles demonstrated a significantly higher bactericidal activity in comparison to bulk CuO microparticles. The spores however showed more resistance towards nanoparticle-mediated sporicidal activity. Effective spore decontamination by nanoparticles occurred in growth media but not in saline media. A “germinate and kill mechanism” is proposed to be operational in LB media.

## Experimental

### Synthesis of CuO nanorods and multi-armed NPs

CuO nanorods (PS2) were synthesized at room temperature by a wet-chemical synthesis of precursor Cu(OH)_2_ nanorods followed by their thermal decomposition (Supporting Information File 1). CuO multi-armed nanoparticles were synthesized by an electrochemical route as described earlier [16].

#### Preparation of *B. anthracis* vegetative cells and spores

*B. anthracis* and *E. coli* bacteria were grown in nutrient broth in an incubator shaker at 37 °C overnight and were used for evaluation of nanoparticles in broth culture test. Standard cultures of the bacteria *B. anthracis* Sterne and *E. coli* were sourced from the High Containment Facility of Microbiology Division, DRDE Gwalior. Various molecular and biochemical tests were performed to confirm the status of each of the cultures. For culturing *B. anthracis* Sterne vegetative cells and *E. coli*, single colonies were taken from nutrient agar plate containing the bacteria and inoculated into 5 mL Luria–Bertani broth (LB broth Ameresco, USA). The inoculated broth was then allowed to grow overnight at 37 °C with constant shaking at 150 rpm. While for culturing *B. anthracis* spores, 100 μL of *B. anthracis* Sterne culture was spread plated on to modified G media and allowed to incubate at 37 °C for a week, to facilitate spore formation.

#### Antibacterial test

Test suspension was prepared by mixing 100 μL of respective bacterial cultures (cell counts varied in the range from 1.25 × 10^4^ CFU/mL to 3 × 10^7^ CFU/mL for *B. anthracis* and from 1.3 × 10^7^ CFU/mL and 2.3 × 10^7^ CFU/mL for *E. coli*) or bacterial spores (7 × 10^4^ CFU/mL and 4 × 10^5^ CFU/mL) with a set dose of CuO nanoparticle suspension. Then the volume is increased to 5 mL by using physiological saline (0.85% aqueous solution), an isotonic media for bacteria. Similar 5 mL of bacterial suspension was prepared without the addition of the nanoparticles as negative control to assess the viability of bacteria in saline during test period. In germinate and kill tests LB broth was prepared by adding 1 g tryptone plus 0.5 g yeast plus 265 mg NaCl to distilled water to prepare 10 mL LB broth in saline. All incubation experiments were performed in an incubator shaker at 37 °C and constant shaking at 150 rpm to ensure maximum contact between CuO nanoparticles and bacterial cells and also to prevent settling of CuO NPs. From test samples, 100 µL were withdrawn and plated on nutrient agar plates at regular time intervals by using the spread plating technique. The plates were then incubated overnight at 37 °C, afterwards colonies were counted.

#### Characterization

The synthesized nanometer-scaled CuO powders were characterized by using a Quanta 400 environmental scanning electron microscope (ESEM), equipped with EDAX energy-dispersive X-ray analyzer for particle shape, size and elemental composition. Before analysis samples were mounted on brass stubs with the help of double sided conductive carbon adhesive tape and coated with gold in a JEOL JFC-1100 sputter coating unit for 10 min. For phase analysis XRD spectra were taken as described in Supporting Information File 1.

## Results and Discussion

### Structure and composition of CuO nanorods (PS2) and CuO multi-armed nanoparticles (P5)

**Figure 1:**
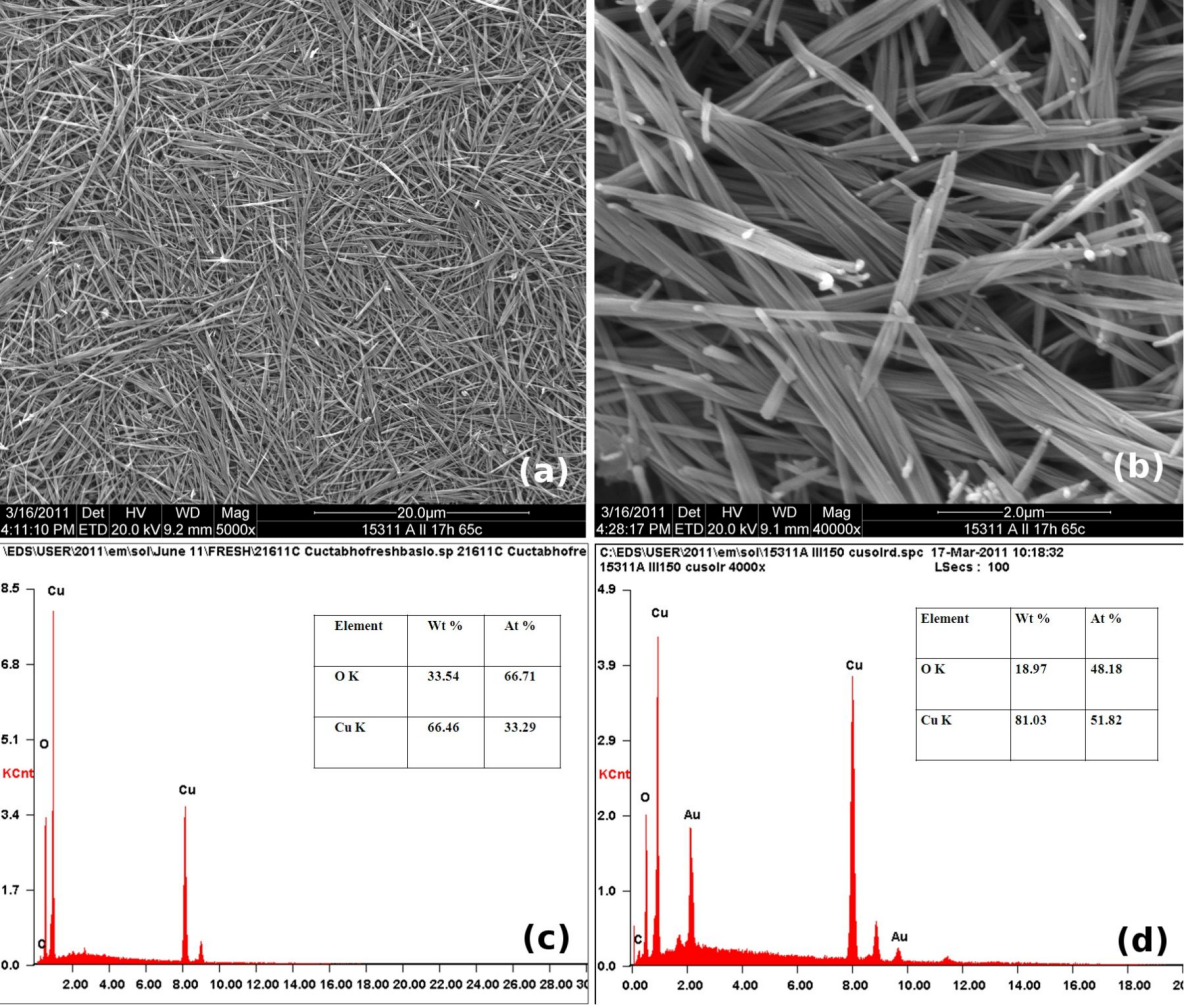
SEM micrograph of CuO nanorods (PS2) at a) 5000× and b) 40000×. c) EDX spectrum of the as-synthesized precursor of PS2. d) EDX spectrum of PS2.

SEM/EDAX reveals the formation of several micrometer-long nanorod bundles with unit rod diameters of about 100 nm and a Cu/O ratio of 1:1 for the PS2 samples prepared by calcinations of the wet-chemically synthesized precursor at 150 °C. Figure 1a and Figure 1b show SEM micrograph of PS2 at 5000× and 40000× respectively. Uniformly formed nanorod bundles that have individual rod diameters of 40–80 nm (average 60 nm with a standard deviation of 9.74 nm) and rod lengths of 4 µm to 11 µm (average 8 µm with a standard deviation of 1.87 µm) constitute PS2. The EDX spectrum (Figure 1d) of PS2 indicates the formation of a CuO phase with a Cu/O ratio of 1:1. On the basis of the EDX spectra it is concluded that the precursor was Cu(OH)_2_ (Figure 1c) which converts into CuO after calcination at 150 °C (Figure 1d). The XRD spectrum (Figure S1a, Supporting Information File 1) further confirms formation of a single phase in which all the peaks can be indexed for the crystal planes of monoclinic CuO (confirmed by JCPDS card match) with a particle size of 8.3 nm (Debye–Scherrer equation).

The electrochemically synthesized product, P5, was nanometer-scaled powder of copper(II) oxide composed of highly branched nanoparticles [16]. Briefly, SEM data (Figure S2a, Supporting Information File 1) show that multi-armed nanoparticles were made of 500–1000 nm long radiating nano-spicules with a Cu/O ratio of around 1:1 determined by EDX analysis (Figure S2b, Supporting Information File 1). The XRD spectrum of multi-armed nanoparticles (P5) (Figure S1b, Supporting Information File 1) confirms the formation of monoclinic single phase CuO with particles sizes of 10.7 nm (Debye–Scherrer equation). The bulk CuO (qualigens) consists of spheroidal micrometer-scaled particles up to 100 µm size having rough surfaces [16].

### Antibacterial test against *B. anthracis* vegetative cells

**Figure 2:**
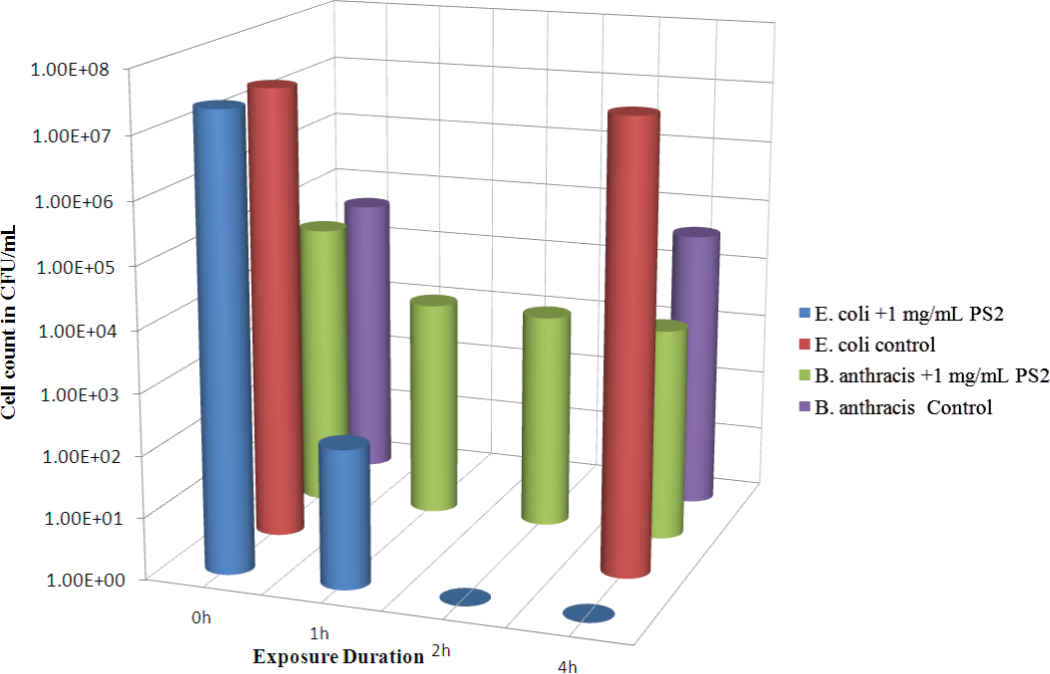
Time dependent bactericidal activity of CuO nanorods (PS2) at a dose of 1 mg/mL against *B. anthracis* vegetative cells and *E. coli* cells in saline media.

The time dependent antibacterial efficacy of CuO nanorods (PS2) against *B. anthracis* bacterial cells and *E. coli* cells at a dose of 1 mg/mL is given in Figure 2. The logarithmic plot of bacterial count against exposure duration shows a reduction in bacterial cell count with an increase in exposure time up to 4 h for both *B. anthracis* and *E. coli* cells. The bar graph shows that 4.5 × 10^4^ CFU/mL *B. anthracis* cells were reduced by 92.17% to 3520 CFU/mL within 1 h of treatment. In fact a reduction larger than 91% occurred within the first 30 min of treatment (data not shown). A reduction of 93.31% occurred after 4 h. The untreated control cells (*B. anthracis*) kept in saline alone under identical conditions showed no adverse effect of saline on the cell viability. A mere 13.33% reduction in the control set cell count may be treated as natural. PS2 NPs demonstrated an even better bactericidal action against *E. coli* at a dose of 1 mg/mL, with which 100% of 2.3 × 10^7^ CFU/mL of bacteria were killed within 2 h of treatment. In fact a reduction of 99.999% took place within the first hour of treatment. In comparison the control *E. coli* set showed only 14.8% reduction in the cell count during the total test period of 4 h. For untreated control sets only the initial count (0 h) and the final count (4 h) were measured for all tests. It is clear from the graph that PS2 NPs show higher a bactericidal action against non-sporogenic bacteria *E. coli* in comparison to *B. anthracis* vegetative cells. The probable reason is discussed later.

**Figure 3:**
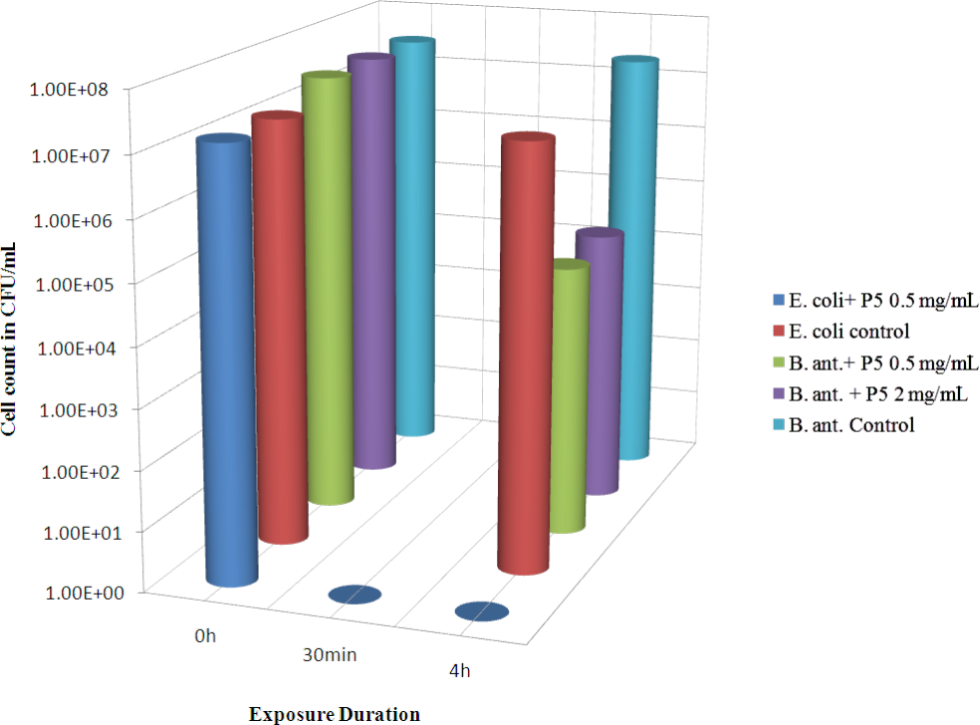
Time dependent bactericidal activity of CuO multi-armed NPs (P5) at doses of 0.5 and 2 mg/mL against *B. anthracis* vegetative cells and at a dose of 0.5 mg/mL against *E. coli* cells (both in saline).

Figure 3 represents the antibacterial activity of electrochemically synthesized CuO multi-armed NPs (P5) at two different concentrations of 0.5 mg/mL and 2 mg/mL against *B. anthracis* vegetative cells and *E. Coli* bacteria. The data show that 3 × 10^7^ CFU/mL *B. anthracis* cells were reduced to 3.64 × 10^4^ CFU/mL that is by 99.88% within 4 h exposure to 0.5 mg/mL dose. Even with higher P5 doses of 2 mg/mL similar reduction of 99.85% was observed. In comparison the untreated cells showed a reduction of 26% within 4 h. With *E. coli* the result was even more remarkable where 1.4 × 10^7^ bacteria were killed within 30 min of exposure to 0.5 mg/mL of P5 compared to 16% reduction in 4 h in the untreated set. It is clear that both the CuO NPs show stronger antibacterial action against nonsporigenic *E. coli* compared to spore forming *B. anthracis* vegetative cells. It is also evident that P5 NPs have stronger antibacterial activity compared to PS2 NPS.

The higher bactericidal activity of CuO multi-armed nanoparticles (P5) compared to CuO nanorods (PS2) could originate from differences in size and shape of the nanorods and the multi-armed nanoparticles coupled with a poor dispersion of PS2 compared to P5. Visibly P5 appeared to be a fine powder whereas PS2 formed thin flakes. SEM (Figure 1a and Figure 1b) shows the nanorods of PS2 stacked together in form of bundles, which in turn form thin flakes (Figure S3a, Supporting Information File 1) unlike P5, which does not form entangled structure. During dispersion experiments PS2 was settling within 15 minutes of free standing even after 5 min sonication. The settling time has been measured for free standing suspension of nanorods by visual inspection after dispersion of 1 mg/mL CuO nanorods in water with or without sonication. The SEM data (Figure S3a–d, Supporting Information File 1) for CuO nanorods sonicated for 0, 2, 5 and 10 min show that the dispersion of nanorods improves with an increase of the duration of sonication (Supporting Information File 1). The bactericidal activity difference between these two CuO nanoparticles could also be due to the difference in their shapes. Sonicated PS2 particles were partially aggregated nanorods, whereas sonicated P5 particles were spear shaped with one tapered end. Most likely the tapered spears of P5 penetrate easily into the bacterial cell on collision causing more cellular damage and cell death compared to the nanorods. The shape of the nanoparticles is known to be an important contributor in their cellular uptake [21]. Recently it has been found that conversion of three-dimensional polystyrene nanospheres to a two-dimensional nanodisc shape offers a larger contact surface with cell membranes and generates less impact during their interaction, which leads to a binding that is limited to the cell membrane with very limited penetration and accumulation inside the cell [22].

**Figure 4:**
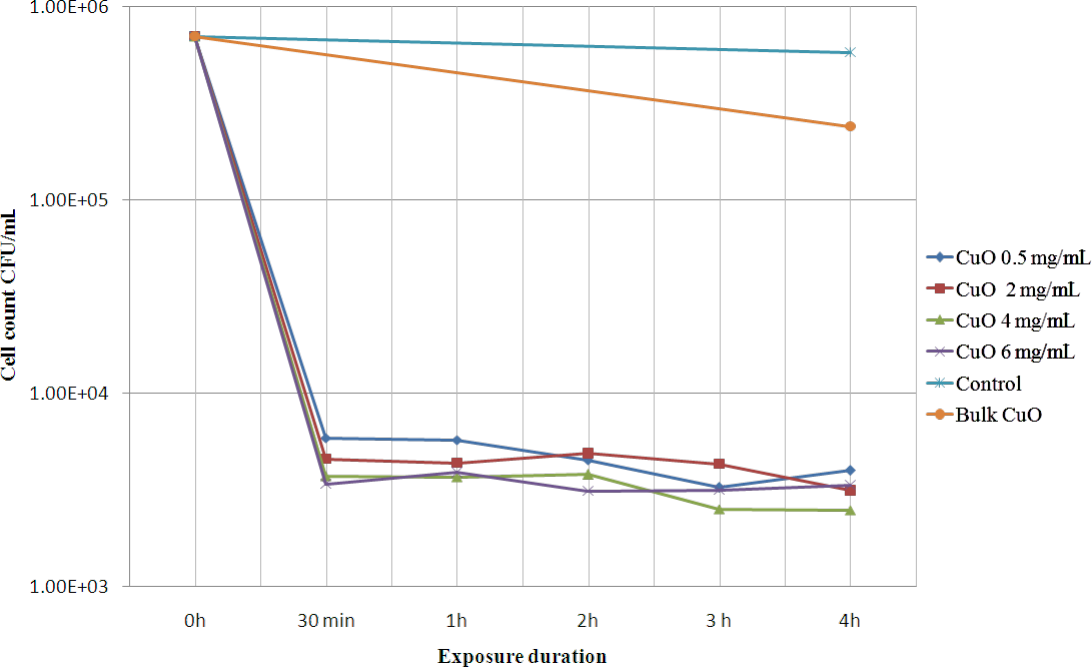
Dose and time dependent bactericidal activity of CuO multi-armed nanoparticles (P5) against *B. anthracis* vegetative cells in saline media compared with bactericidal activity of bulk CuO (commercial).

The dose- and time-dependent bactericidal activity of multi-armed CuO nanoparticles (P5) against 7.0 × 10^5^ CFU/mL *B. anthracis* vegetative cells at dose range of 0.5 to 6 mg/mL is shown in Figure 4. The graph also compares bactericidal activity of P5 nanoparticles to that of bulk CuO microparticles along with a negative control. The negative control represents a survival of the cells in saline media during test period in absence of any CuO. It can be seen from the graph that steep reduction of more than 99% in cell count occurred within first 30 min of exposure to CuO nanoparticles (P5). Interestingly even 0.5 mg/mL dose of the NPs was capable of killing over 99% of *B. anthracis* vegetative cells. Afterward, up to 4 h the rate of decrease was not that high, even for higher dosage up to 6 mg/mL P5 NPs. In terms of absolute data after exposure to 0.5, 2, 4 and 6 mg/mL P5 NPs a decrease in cell count of 99.15%, 99.34%, 99.46% and 99.51%, respectively, occurred within 30 min and a decrease in cell count of 99.42%, 99.55%, 99.64%, 99.52%, respectively, occurred within 4 h. In comparison the bulk CuO particles killed only 65.71% of the cells within 4 h at 2 mg/mL dose. These antibacterial tests were repeated several times and 100% bactericidal action of P5 was rarely observed. A small fraction of bacteria remained viable even after 24 h of exposure to nanoparticles. The increase in lethality of nanoparticles due to increase in the dose of P5 NPs from 0.5 to 6 mg/mL is small regarding the fact that the to the dose increased by about 12 times.

**Figure 5:**
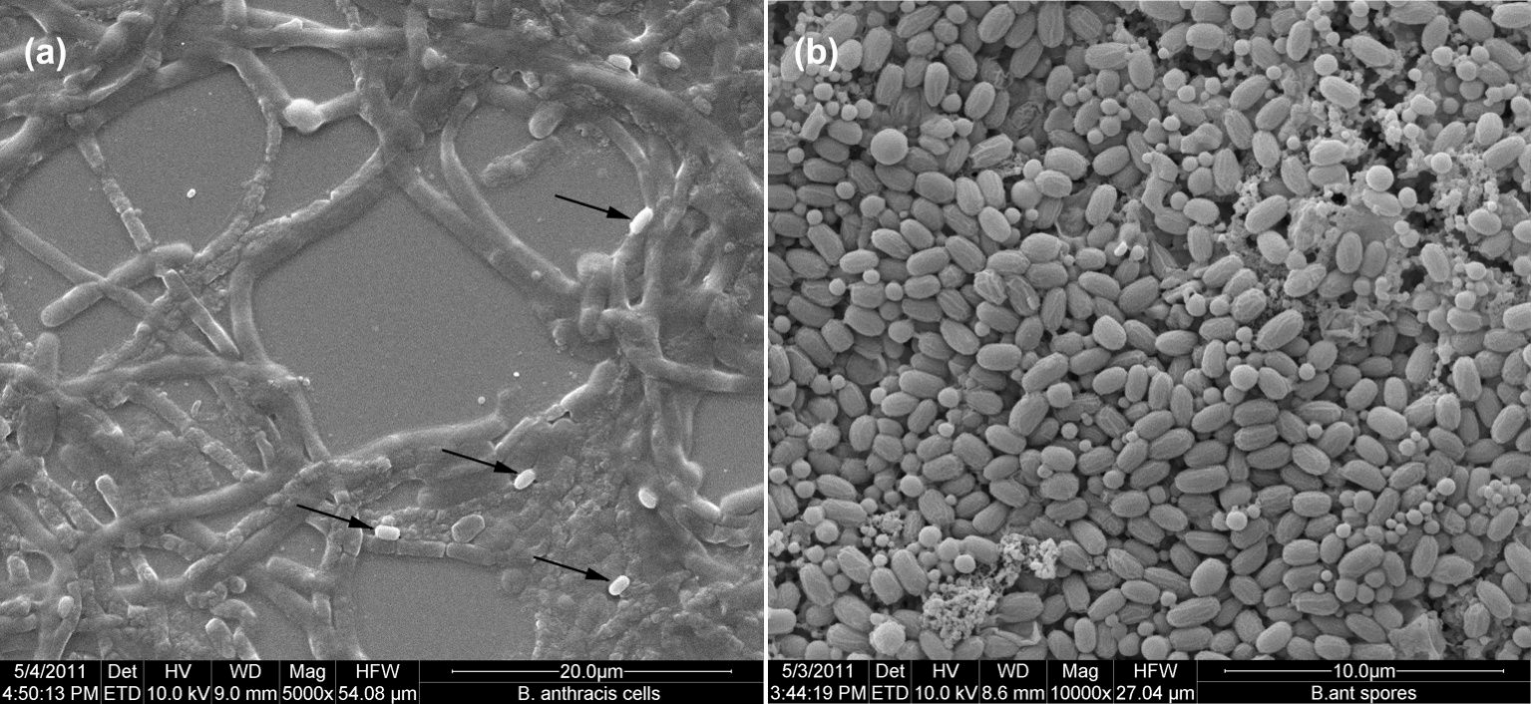
SEM micrographs of a) *B. anthracis* control cells at 5000× the arrows show occasional spores population present in the cell culture. b) *B. anthracis* control spores formed in G-media after seven days of incubation at 10000× magnification.

During electron microscopic investigation of bacterial cells occasional spores among the numerous cells were observed (pointed at by the arrow-heads in Figure 5a). Figure 5b shows *B. anthracis* spores prepared by the method described in section Experimental. The SEM micrographs indicate the presence of a small fraction of spores in vegetative cell cultures. Probably this spore fraction resists bactericidal action of nanoparticles in test suspension and later on germinates in agar culture plate. Occasionally 100% deactivation of *B. anthracis* cells also occurred in some of the tests for cell counts as high as 2.6 × 10^5^ CFU/mL by 1 mg/mL CuO multi-armed nanoparticles within 2 h of exposure. These results further strengthen the probability of presence of a small nanoparticle resistant spore fraction in *B. anthracis* vegetative cell culture in most of the batches with the exception of few.

The nanoparticles probably kill the cells by damaging the cell membrane mechanically, leading to leakage of cellular content. SEM observations discussed in later paragraphs support this probability. The broad spectrum of antibacterial activity of CuO nanoparticles against gram-positive and gram-negative bacteria and higher bactericidal efficacy of the spear-shaped CuO nanoparticles (P5) compared to rod-shaped CuO nanoparticles (PS2) also supports the possible mechanical damage to be the main cause for bacterial killing. Although dissolved copper ions may also contribute to some extent in bactericidal action [16,23].

An experiment was conducted to ascertain the effect of an accelerated rate of mixing on the biocidal activity of CuO NPs (P5). Table 1 shows the data of parallel antibacterial tests conducted in incubator shaker bath at 150 rpm and ultrasonic cleaning bath (Spectralab, India). It was expected that accelerated mixing in ultrasonic bath would lead to higher mechanical damage to cells in test suspension carrying bacterial cells and CuO NPs due to an increased frequency of collisions between the two. The results showed that bacterial cells were highly susceptible to CuO NPs as 99.6% of the cells were killed within first 5 min of exposure to 2 mg/mL P5 NPs in both exposures. It seemed hard to kill the remaining 0.4% fraction even after 30 min. This little fraction could be consisted of spores and not of cells. Because of the very high killing efficiency of P5 NPs even shaking at 150 rpm was equally effective as the sonicating bath treatment. Control cells in both of the sets show a similar viability. It appears that the sonicating bath treatment did not cause any extra damage to the cells when compared to shaking at 150 rpm. Probably the cells and nanoparticles collide to each other due to Brownian motion and electrostatic attraction between positively charged CuO nanoparticle surfaces and negatively charged bacterial cells. Though no independent measurement of zeta potential has been carried out in the present study, it has been reported that CuO nanoparticles in nanofluid dispersion carry a positive zeta potential, i.e., a positive overall surface charge below pH 9.2, which is the isoelectric point of the CuO nanofluid [24]. In the current study the pH has not been adjusted but it has been near 7. Once in contact the reaction between the bacterial cells and the nanoparticles results in a reduction of Cu^2+^ to Cu^+^ at the surface of the nanoparticles. The probable oxidation of bacterial cell walls, mechanical damage and leakage of cytoplasm could be the main reason of cell death along with other physicochemical reactions resulting from CuO NP and copper ion uptake. To corroborate this phenomenon an antibacterial test was carried out for a high cell count and low nanoparticle (P5) ratio so that viable cells should not be occluded by excess nanoparticles after the treatment. In this test 3.9 × 10^6^ CFU/mL *B. anthracis* cells were exposed to 0.1 mg/mL of nanoparticles for 4 h in an incubator shaker at 150 rpm at 37 °C. After the test supernatant and settling fractions of the antibacterial test suspension were collected and centrifuged at 10,000*g*. SEM analysis of the supernatant fraction and the settling fraction reveals presence of irregularly shaped cell debris (Figure S4a, Supporting Information File 1), which probably resulted from mechanical damage, along with damaged cellular chains showing necking and fracture formation as well as pitting on the cellular surface (Figure S4b, Supporting Information File 1). This is unlike untreated *B. anthracis* cells, which exhibit intact cell chains with smooth slime like thin covering (Figure S4c, Supporting Information File 1). Accumulation of CuO nanoparticles at pitted sites (Figure S4b, Supporting Information File 1) is also notable with less prominence of a slime like layer in treated cells compared to the control cells.

**Table 1:** Antibacterial activity of multi-armed CuO NPs (P5) against *B. anthracis* vegetative cells at 2mg/mL of P5 in saline – under sonication in ultrasonic bath or shaking at 150 rpm in an incubator shaker (at 35 °C).

exposure time (min)	*B. anthracis* cells (CFU/mL)			
	control cells (incubator shaker)	cells with P5 NPs (incubator shaker)	control cells (sonicating bath)	cells with P5 NPs (sonicating bath)

0	1.25 × 10^4^	1.25 × 10^4^	1.25 × 10^4^	1.25 × 10^4^
5	1.38 × 10^4^	50	1.3 × 10^4^	50
15	1.3 × 10^4^	30	1.26 × 10^4^	30
30	1.2 × 10^4^	30	1.28 × 10^4^	30

Recently Yang Li et al. have established that CuO NPs do not produce reactive oxygen species (ROS) even after 48 h of irradiation with UV light. Therefore, photocytotoxicity plays no role in the strong bactericidal effects shown by CuO NPs [23]. However they found a very small equilibrium ionisation of CuO NPs to Cu^2+^ ions (30 µg/L Cu^2+^ ions in a 5 mg/L CuO NP suspension) in their experiment and proposed that the strong *E. coli* inactivation could be caused by the miniscule quantity of released ions. However when *E. coli* cells were exposed to a CuO NP suspension that was kept in a dialysis bag to ensure that only ions were released in the bacterial culture the bactericidal activity reduced significantly in comparison to the CuO NP suspension in direct contact with *E. coli* cells [16]. Contrary to Li’s work, Guy Appelrot et al. have found the antibacterial activity of CuO nanoparticles to be due to the generation of ROS by the NPs attached to the bacterial cells, which in turn enhanced the intracellular oxidative stress [25]. By using electron microscopy they also detected the presence of small nanoparticles of CuO penetrated in the cells. Apparently a combination of mechanisms operates during the bactericidal action of CuO NPs including mechanical damage, NP penetration in to the cell, ROS generation by attached particles and copper ion formation. This multi-pronged antibacterial action will make it difficult for bacteria to develop a resistance against CuO NPS. It can be noted from Table 1 that the remaining 0.4% organisms show a strong resistance to CuO NP mediated deactivation in both test sets and a countable number of organisms was present even after 30 min. This observation further strengthens the possibility that this viable fraction represents thick walled spores. Moreover, the data show the advantage of using nanometer-scaled CuO over the photocatalytic deactivation of *B. anthracis* cells by nanometer-scaled TiO_2_. Deactivation of comparatively fewer *B. anthracis* cells (1900 CFU/mL) by nanometer-scaled TiO_2_ has been reported earlier in the presence of UVA light at higher doses (75 mg) of nanoparticles [13]. In the present study CuO nanoparticles were found to be highly effectively antibacterial at doses as low as 0.1 to 6 mg/mL and without the assistance of UV light. Unlike TiO_2,_ which is a photocatalyst, the CuO nanoparticles do not need any assistance of light, to attain their decontamination performance. Therefore these nanoparticles can be used for decontamination of indoor settings and for decontamination of water without UV light irradiation.

### Sporicidal activity against *B. anthracis* spores

**Figure 6:**
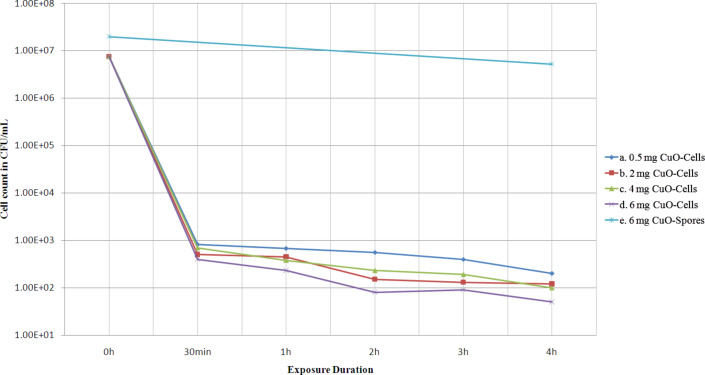
Antibacterial activity of multi-armed copper oxide NPs (P5) against *B. anthracis* vegetative cells and *B. anthracis* spores at various time intervals. The control set has shown a variation below 20% during the test period (4 h).

To verify the hypothesis that the small viable fraction observed in antibacterial tests of CuO NPs against *B. anthracis* cells could be spores in test cultures, an antibacterial test was carried out comparing the antibacterial action of P5 nanoparticles against *B. anthracis* vegetative cells and *B. anthracis* spores (Figure 6). The figure represents data for antibacterial test of P5 NPs against *B. anthracis* vegetative cells and spores at various time intervals with concentrations of CuO NPs of 0.5 to 6 mg/mL. An almost uniform decrease of 99.99% in *B. anthracis* cells from the initial count of 7.5 × 10^6^ CFU/mL occurred within 30 min of exposure to 0.5, 2, 4 and 6 mg/mL P5 nanoparticles, respectively. After 4 h of exposure the reduction was 99.997%, 99.998%, 99.998% and 99.999% for 0.5, 2, 4 and 6 mg/mL P5 NPs respectively, leaving behind a small viable fraction. However, during the four hour exposure period *B. anthracis* spores exhibited a 73.75% reduction in initial spore count of 2.0 × 10^7^ CFU/mL, which is not significantly different from reduction in negative control set spore count that ranged between 30 and 60% in different tests. Repeated experiments gave similar findings. The data support the assumption that *B. anthracis* vegetative cells are susceptible to deactivation by P5 NPs and the residual counts (Figure 4 and Figure 6) that survive prolonged treatments could be of small percentage of spores among the vegetative cells. Table 2 shows comparison of sporicidal activities of PS2 and P5 against *B. anthracis* spores within 4 h exposure in saline media. The table shows that spores are very resistant to deactivation by both the nanoparticles.

**Table 2:** Antibacterial activity of multi-armed copper oxide nanoparticles (P5) and CuO nanorods (PS2) against *B. anthracis* spores with 10 mg/mL of CuO-NPs in distilled water.

exposure time (h)	*B. anthracis* spores (CFU/mL)		
	control	P5	PS2

0	7 × 10^4^	7 × 10^4^	7 × 10^4^
4	4.8 × 10^4^	4.2 × 10^4^	4.5 × 10^4^

The exceptionally high resistance of *B. anthracis* spores compared to high susceptibility of vegetative cells to CuO nanoparticles (P5) can be explained in terms of morphology and composition of cells and spores. Cells, because of their thin protective outer layer and relatively large fluidity of cytoplasm, are more prone to mechanical damage by rod-like (PS2) and spike-like (P5) nanoparticles. In comparison, for bacterial spores, because of the complex shell-like structure of endospores composed of several thick protective barrier layers coupled with a nearly desiccated core with minimal metabolic activity, a high intrinsic resistance is expected. The saturation of spore DNA with a group of unique proteins called α/β-SASP (small acid-soluble spore proteins) and the high mineral content in the form of various divalent cations, in particular Ca^2+^ may also be contributing factors [26–27].

### Germinate and kill strategy for spore deactivation

**Figure 7:**
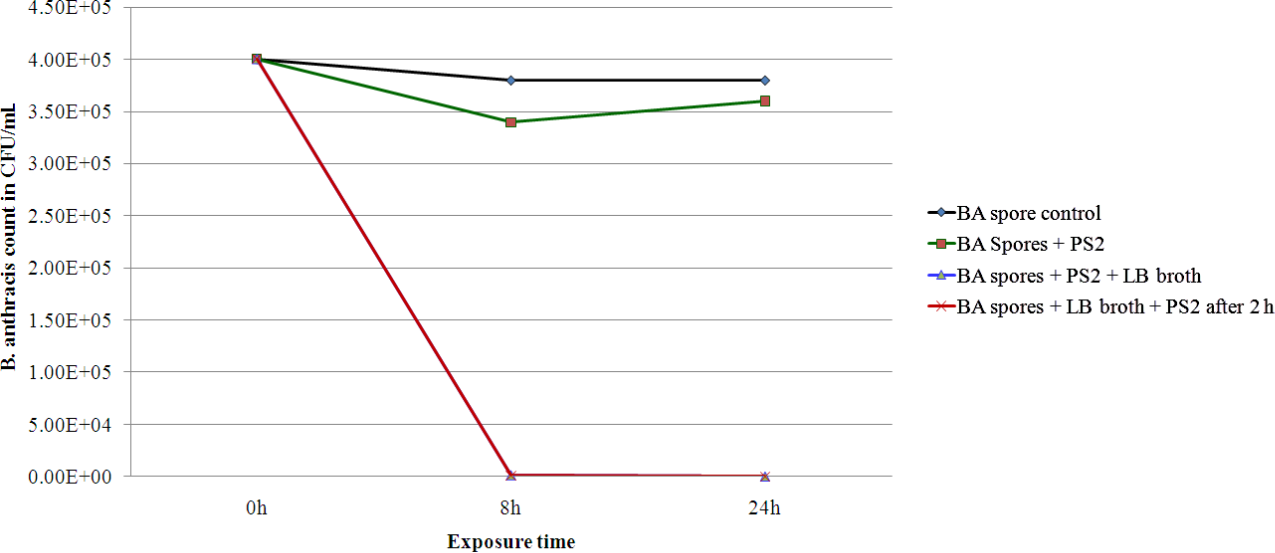
Antibacterial activity of CuO nanorods (PS2) against *B. anthracis* spores in presence and absence of growth media namely LB broth.

Figure 7 shows that in LB broth nanoparticles kill more than 99.6% of 4 × 10^5^ CFU/mL *B. anthracis* spores within 8 h and over 99.99% of bacteria within 24 h at a nanoparticle dose of 2 mg/mL. Similar results were obtained for the spores incubated in LB broth for 2 h at 37 °C before the addition of PS2. In comparison the saline plus PS2 set showed less than 20% reduction only in the spore count. Although CuO nanoparticles showed a poor sporicidal activity in saline media, with over 99.99% deactivation of spores their activity in LB growth media is significant.

**Figure 8:**
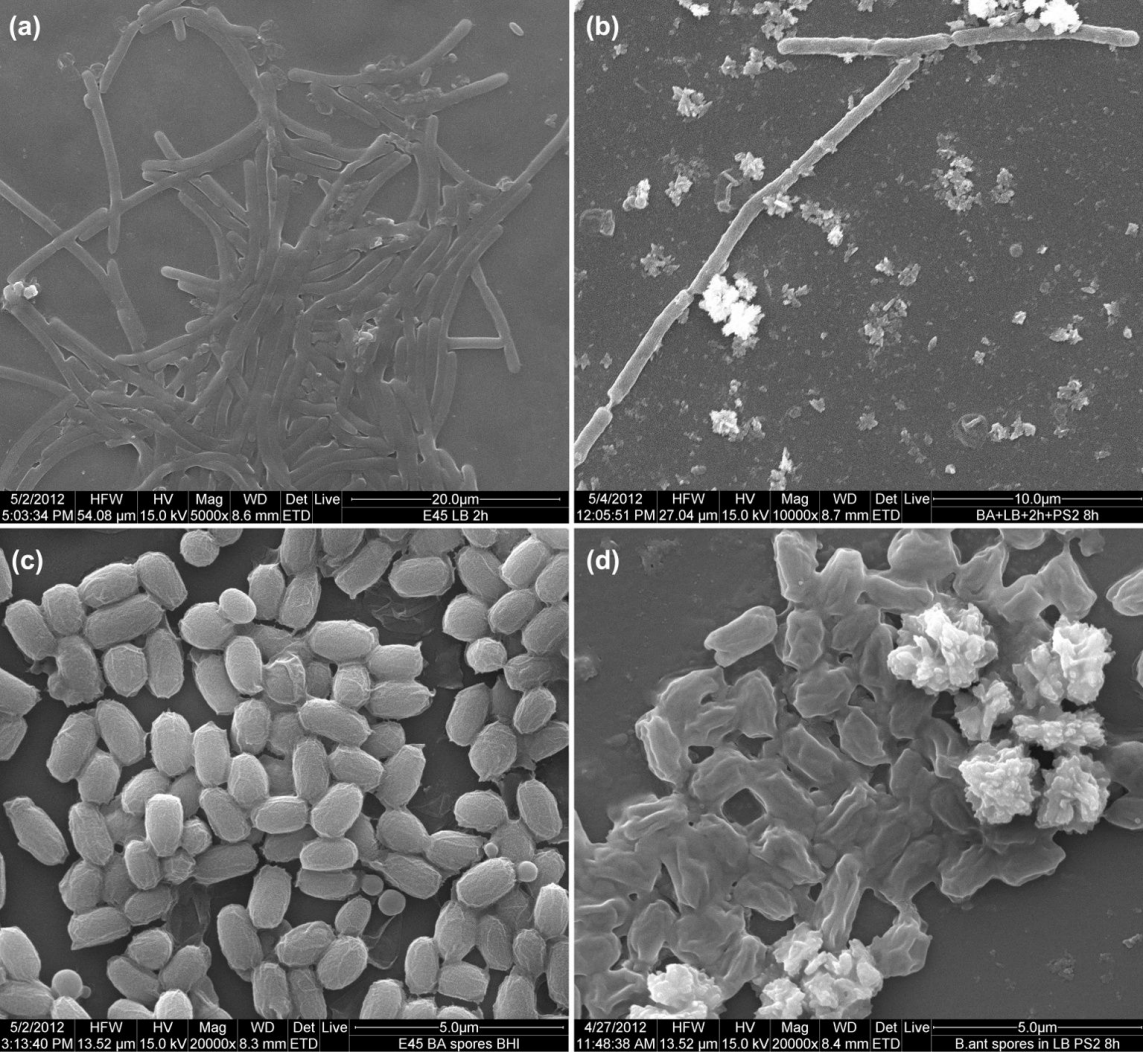
SEM micrographs of *B. anthracis* spores. a) After 2 h of incubation in LB broth, 5000×. b) After 2 h of incubation in LB broth then PS2 addition and 8 h of incubation overall, 10000×. c) Control untreated spores, 20000×. d) Spores in LB broth plus PS2 after 8 h of incubation, 20000×.

Apparently, a “germinate and kill” mechanism was operational in presence of growth media. First, the spore germination was triggered by germinants present in the growth medium, and later the germinated spores were killed by the CuO nanoparticles. The SEM images (Figure 8) of the control spores and spores incubated in LB broth with different treatment-conditions support this deduction. The SEM micrographs show that spores germinate and form short cellular chains with smooth cell walls, if kept in LB broth alone for 2 h (Figure 8a). Whereas, PS2 nanoparticle addition and a further incubation of up to 8 h, result in a reduction in the number of cells (Figure 7) and also a reduction in their diameter by about 25% in the treated group (Figure 8b). The cellular surfaces and inter-cellular junctions exhibit damage and necking respectively. The observation corroborated well with plating data presented in Figure 7. On the other hand the spores treated with PS2 NPs in LB broth show a soft and pliable appearance (Figure 8d) when compared with untreated spores (Figure 8c). Unlike during the prior incubation in growth media, this cellular structure did not form when bactericidal nanoparticles were present in growth medium. These soft and pliable spores could most probably be germinated spores, which are susceptible to the bactericidal activity of CuO nanoparticles. In short, copper oxide NPs are capable of deactivating *B. anthracis* spores in the presence of growth media but not in saline media. Also, though the presence of nanoparticles allows for the germination of spores, it did not allow vegetative cell outgrowth or cellular division. A single germinant plus CuO nanoparticle combined formulation that can be used for decontamination of *B. anthracis* spores holds a good potential.

Our findings regarding the difference between the bactericidal and the sporicidal potential of CuO nanoparticles are in accordance with the existing knowledge. Bacterial spores of the genera *Bacillus* and *Clostridium* have been widely studied and are invariably the most resistant of all types of bacteria to the antiseptics and disinfectants [28–32]. It is well known that many biocides are bactericidal or bacteristatic at low concentrations for the vegetative cells of *Bacillus* species, but that higher concentrations may be necessary to achieve a sporicidal effect (e.g., for glutaraldehyde and CRAs). Nonetheless, certain bactericides like alcohol, phenolics, quaternary ammonium compounds, and chlorhexidine lack a sporicidal effect even at high concentrations [29,33]. The cause apparently lies in the inherent high resistance of spores, which are formed by the bacteria as a measure to survive in an adverse physical/chemical environment. An alternative strategy to decontaminate the spores could be to germinate them and target the germinated cells. Present study underscores that the *B. anthracis* vegetative cells are nearly as susceptible to CuO nanoparticle as other cellular bacteria such as *E. coli* evaluated in this study. The effective killing of *B. anthracis* vegetative cells by CuO nanoparticles elicit that these nanoparticles can be part of a decontaminant system constituted of spore germinants plus nanoparticle bactericide. Our group is further focusing on the strategy of germinating the spores and the subsequent in-situ killing of the germinated *B. anthracis* cells by using single germinant–nanoparticle composition.

## Conclusion

Different morphologies of nanometer-scaled CuO, i.e., nanorods and multi-armed NPs, were synthesized by either using a simple wet-chemical route or an electrochemical deposition route. The nanoparticles have shown a strong bactericidal potential against *B. anthracis* vegetative cells that was comparable to their activity against gram-negative *E. coli* bacteria. Both of these nanoparticles exhibited 91–99.99% killing in a dose range between 0.5 and 2 mg/mL within 30 min of exposure for cell counts in the range between 4.5 × 10^4^ and 3 × 10^7^ CFU/mL. However, increasing the exposure duration up to 4 h or the dose up to 6 mg/mL showed a minuscule gain in bactericidal activity in most of the antibacterial tests. Whereas 100% bactericidal activity was demonstrated against gram-negative *E. coli* bacteria when 2.3 × 10^7^ CFU/mL cells were killed within 2 h of exposure to 1 mg/mL CuO nanorods (PS2) and 1.4 × 10^7^ CFU/mL cells were killed within 30 min of exposure to 0.5 mg/mL CuO multi-armed nanoparticles (P5). Also 1 × 10^4^ *B. anthracis* cells were killed within 5 min of exposure to 2 mg/mL CuO NPs (P5). The nanoparticles were proved to be far more efficient than the bulk CuO as exemplified by a 99.55% reduction in count of *B. anthracis* compared to 65.71% reduction by bulk CuO under identical conditions. The significant bactericidal activity of CuO NPs has been apparently caused by a combined mechanism of mechanical damage by spear- and rod-like nanoparticles coupled with the oxidation of the cellular surface and the generation of Cu^2+^ ions. However, the spores show a strong resistance against nanoparticles-mediated inactivation in saline media. Although in presence of growth media a 99.6% reduction in spore count from original 4 × 10^5^ CFU/mL within 8 h and 99.99% reduction within 24 h of exposure to CuO nanorods could be obtained. This sporicidal action is due to a “germinate and kill mechanism” operational in LB growth medium with nanoparticles. This work further confirms our earlier finding that CuO nanoparticles act as a broad-spectrum antibacterial agent with excellent bactericidal and sporicidal activity against *B. anthracis* vegetative cells and germinated spores. Being noncorrosive, CuO NPs can find a potential application in the solid-phase decontamination of equipment and surfaces against the deadly warfare agent *B. anthracis*.

## Supporting Information

File 1 Additional experimental data.
