# Supplementary material for: Antimicrobial properties of CuO nanorods and multi-armed nanoparticles against B. anthracis vegetative cells and endospores
Source: Beilstein J Nanotechnol. 2014 Jun 5;5:789–800. doi: 10.3762/bjnano.5.91 (PMC4077429; doi:10.3762/bjnano.5.91)
Supplement: File 1 — Additional experimental data. [file Beilstein_J_Nanotechnol-05-789-s001.pdf]

Supporting Information

for

Antimicrobial properties of CuO nanorods and multi-armed nanoparticles against *B. anthracis* vegetative cells and endospores

Pratibha Pandey*[1,] Merwyn S. Packiyaraj[2], Himangini Nigam[2], Gauri S. Agarwal[2], Beer Singh[1, 3] and Manoj K. Patra[4]

[1]Electron Microscopy Division Defence R&D Establishment, New Campus, Jhansi Road, Gwalior, India, [2]Microbiology Division Defence R&D Establishment, New Campus, Jhansi Road, Gwalior, India, [3]Protective Devices Division Defence R&D Establishment, New Campus, Jhansi Road, Gwalior, India and [4]Defence Laboratory, Jodhpur, India.

Email: Pratibha Pandey* - rkpratibha@yahoo.com

* Corresponding author

Additional experimental data

Wet-chemical synthesis of CuO nanorods (PS2)

For the wet-chemical synthesis, 100 mL 0.01 M aqueous $CuSO_4$ (Qualigens-ExelaR) solution containing 25 mL 0.02 M cetyl trimethyl ammonium hydroxide (CTAB, Sigma Aldrich ≥ 98%) aqueous solution as a capping agent and 2.5 mL 30% H_2O_2 (Merck) as an oxidant was prepared by 20 min mixing in a sonicating bath. The $CuSO_4$ was then hydrolyzed by addition of 40 mL 0.5 M aqueous NaOH (Sigma Aldrich ≥ 98%) into the above solution. The final pH was around 12.5. Instantly a coffee brown precipitate formed, which slowly turned into a voluminous pastel blue precipitate. After 2 h the precipitate was washed repeatedly till neutral pH and dried either at 50 °C or calcined at 150 °C.

X-ray diffraction analysis (XRD)

The XRD analysis of the synthesized powders was performed on a PANalytical, X'pertPro XRD instrument using Cu Kα line (wavelength 1.54060 Å). The size determination of the nanoparticles was carried out with the help of the Debye–Scherrer equation. Particle size was determined by Debye–Scherrer equation (Equation 1).

$$D\ (nm) = \frac{0.89\lambda}{B\cos\theta} \qquad (1)$$

Using above equation the average crystallite size, *D*, of the nanoparticles can be determined from the XRD spectrum, where λ denotes the wavelength of the X-rays (1.54Å, Cu Kα), θ is the angle of diffraction and *B* is the full width at half maximum (FWHM) in radian. As can be seen in Figure S1a the high intensity of the (002) and (111) diffraction peaks suggests that the CuO nanorods (PS2) are primarily dominated by (002) and (111) facets.

XRD spectrum of multi-armed nanoparticles (P5) (Figure S1b) confirms the formation of monoclinic single phase CuO with particles size of 10.7 nm (Debye–Scherrer equation). As can be seen in Figure 2c the high intensity of the (002) and (111) diffraction peaks suggests that the CuO multi-armed nanoparticles (P5) were also dominated by (002) and (111) facets. However when compared with CuO nanorods (PS2) diffraction peak for (111) plane has higher intensity than (002) plane.

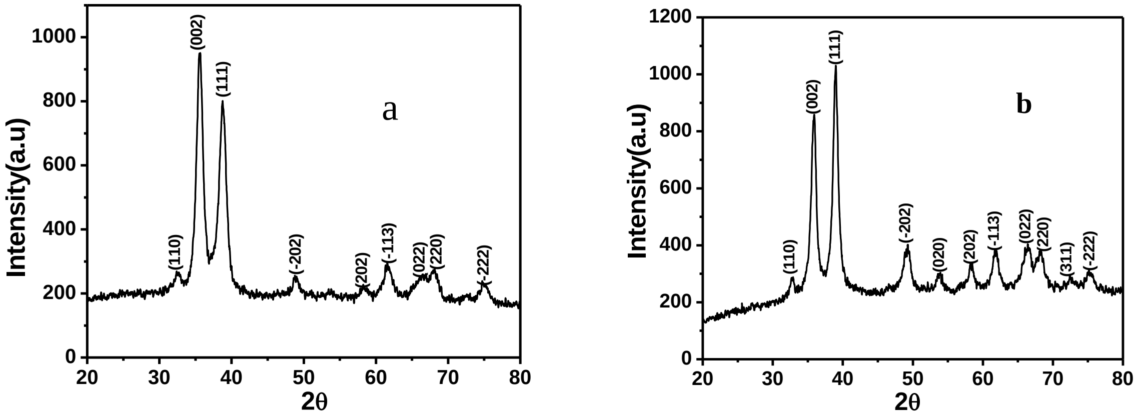

Figure S1: XRD spectrum of a) PS2, b) P5.

Structure and composition of CuO nanorods (PS2)

The morphology and composition of wet-chemically synthesized $Cu(OH)_2$ precursor and CuO nanoparticles prepared by calcinations of $Cu(OH)_2$ at 150 °C was established with the help of SEM/EDX and XRD. During wet-chemical synthesis the initially formed precipitate was composed of precursor $Cu(OH)_2$ nanorod bundles as found by SEM/EDX characterization of the fraction dried at 50 °C. After heating it to 150 °C the $Cu(OH)_2$ phase converts into deep brown CuO nanorod powder due to dehydration. SEM/EDAX reveals the formation of several micrometer long nanorod bundles having unit rod diameters within 100 nm and a Cu/O ratio of

1:2 for the wet-chemically formed blue precipitate dried at 50 °C and 1:1 ratio for the PS2 samples prepared by calcinations of the precipitate at 150 °C.

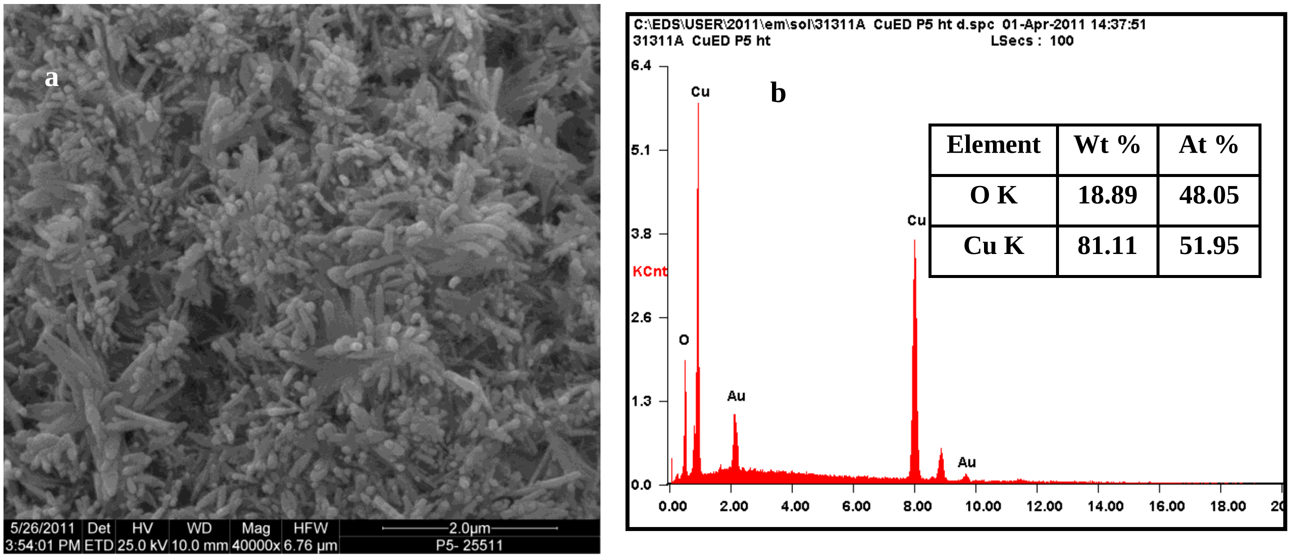

Figure S2: a) SEM micrograph of multi-armed nanoparticles (P5) at 40000×. b) EDX spectrum of P5.

Dispersibility of CuO NPs

The SEM data in Figure S4 for CuO nanorods sonicated for 0, 2, 5 and 10 min show that the dispersion of nanorods improves with an increase in duration of sonication. Homogeneous dispersion of PS2 did not occur without sonication (0 min) micrometer-sized flakes were visible in the suspension. The SEM micrograph in Figure 5a shows that the flakes consist of numerous entangled nanorods. After 2 min sonication a partial dispersion however does occur (Figure 5b) but the particles were completely settled within 10 min of free standing. After 5 min sonication settling occurred within 15 min and after 10 min of sonication the particles took 20 min to settle.

The micrographs in Figure S3c and Figure S3d show separated rod bundle (marked by arrow heads) scattered on the surface of the porous filter membrane. Therefore the lower bactericidal activity of PS2 may result from poor dispersion of entangled nanorods compared to the well dispersed multi-armed nanoparticles, which have been found to be broken in to individual nanospindles or nanospicules even after 1 min of sonication [16].

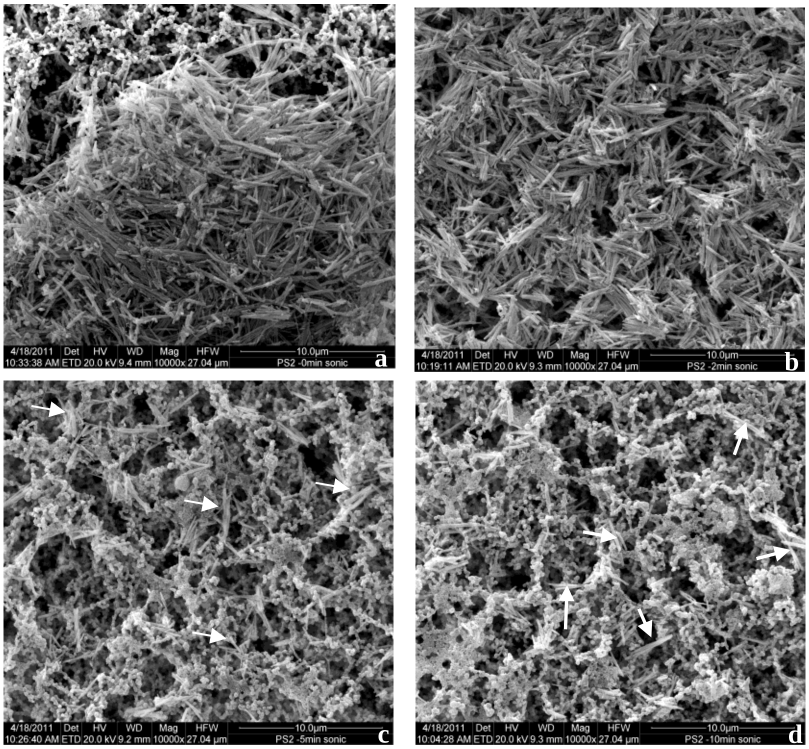

Figure S3: SEM micrographs representing the dispersion behavior of PS2 at 10000×. a) Without sonication, b) after 2 min of sonication, c) after 5 min of sonication, d) after 10 min of sonication. The suspensions were filtered through 0.2 μm pore size nitrocellulose filter membrane seen in the background. The arrow-heads point toward isolated nanorod bundles.

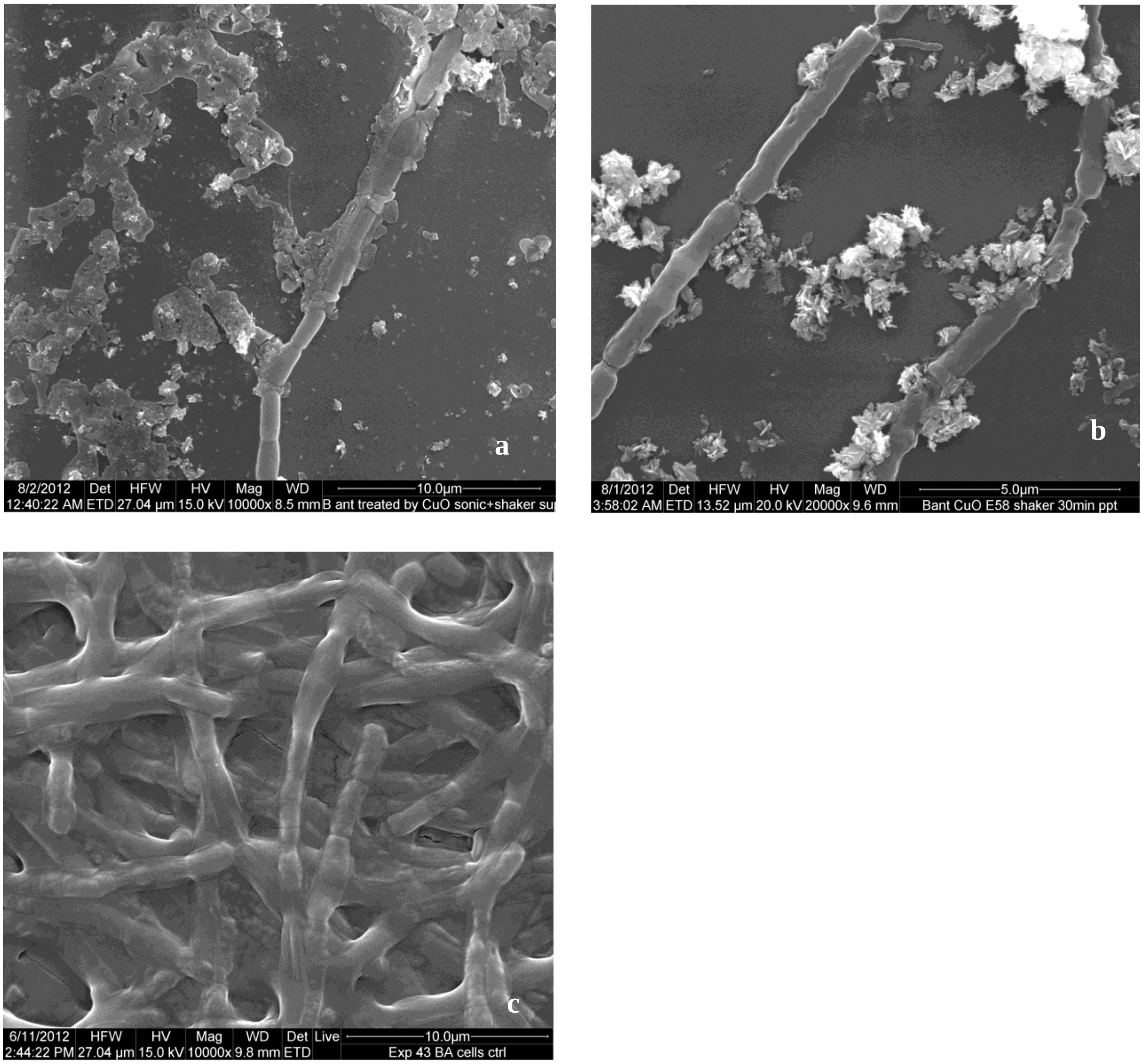

Figure S4: SEM micrographs of antibacterial test suspension. a) Floating fraction, b) settling fraction and c) control *B. anthracis* cells at 10000×, 20000× and 10000×, respectively.
